# The Impact of External Factors on the Epigenome:* In Utero* and over Lifetime

**DOI:** 10.1155/2016/2568635

**Published:** 2016-05-18

**Authors:** Estela G. Toraño, María G. García, Juan Luis Fernández-Morera, Pilar Niño-García, Agustín F. Fernández

**Affiliations:** ^1^Cancer Epigenetics Laboratory, Instituto Universitario de Oncología del Principado de Asturias (IUOPA), HUCA, Universidad de Oviedo, 33006 Oviedo, Spain; ^2^Servicio de Salud Laboral, Instituto Asturiano de Prevención de Riesgos Laborales, 33006 Oviedo, Spain

## Abstract

Epigenetic marks change during fetal development, adult life, and aging. Some changes play an important role in the establishment and regulation of gene programs, but others seem to occur without any apparent physiological role. An important future challenge in the field of epigenetics will be to describe how the environment affects both of these types of epigenetic change and to learn if interaction between them can determine healthy and disease phenotypes during lifetime. Here we discuss how chemical and physical environmental stressors, diet, life habits, and pharmacological treatments can affect the epigenome during lifetime and the possible impact of these epigenetic changes on pathophysiological processes.

## 1. Introduction

The meaning of the term epigenetics has evolved considerably over time. Conrad Hal Waddington coined the term in the 1940s to describe the “causal mechanisms” that give rise to phenotypes from genotypes in the developmental and differentiation processes [1]. Nowadays, the term is used to explain stable heritable chemical modifications to DNA and histones that affect gene expression without altering nucleotide sequence [2]. This new concept has allowed the consideration of a new perspective from which the complexity of many cellular processes such as genetic regulation, cellular development and differentiation, genomic imprinting, embryology, aging and cancer, and other diseases is understood. What is more, epigenetic alterations may occur due to chance or under environmental influence [3]. In the latter case, epigenetics moderates the genetic expression of a trait depending on the prevailing environmental conditions, a phenomenon which could confer an organism with the necessary plasticity to adapt to its environment and the capacity to induce alternative phenotypes from the same genotype through the regulation of gene expression patterns [4].

Environmental epigenetics emerges from the idea that the interaction between the environment and the epigenome may alter the phenotype and might be related to disease susceptibility. And most importantly, these alterations could be transmitted down through generations [5, 6]. The epigenome is at risk of changes and alterations over time, and it will be dependent on internal, external, and/or stochastic factors [7]. In this review we will describe how external factors affect the epigenome and the consequences for health and disease during lifetime. We will discuss recent works on how epigenetic mechanisms, such as DNA methylation, histone posttranslational modifications, and noncoding RNAs, particularly microRNAs, are affected by environmental aspects, such as different chemical and physical environmental stressors, diet, unhealthy habits, and pharmacological treatments. The principal epigenetic mechanisms will be described and we will also discuss how epigenetic alterations caused by external factors could mediate the appearance of disease phenotypes.

## 2. Epigenetic Mechanisms

Chromatin is a highly regulated complex of macromolecules in the nucleus formed by DNA, histone proteins, and RNA. The nucleosome is the basic repetitive unit of chromatin and consists of 147 base pairs around an octamer of the four core histones (H2A, H2B, H3, and H4) [8]. Differences in the degree of compaction determine its structure and complexity which in turn facilitate its differentiation into two functional states: euchromatin or heterochromatin, which are the transcriptionally active and inactive forms, respectively [9]. Epigenetic mechanisms such as DNA methylation and histone modifications participate in the remodeling of chromatin. These changes in chromatin structure are able to modify the accessibility of genes to transcriptional machinery, regulate gene expression during development and differentiation stages, and determine which genes are transcribed [10]. Aberrant profiles of these epigenetic processes may result in mismatches in important signaling pathways that alter various cell functions and may lead to the development of different diseases such as cancer [11].

**Figure 1:**
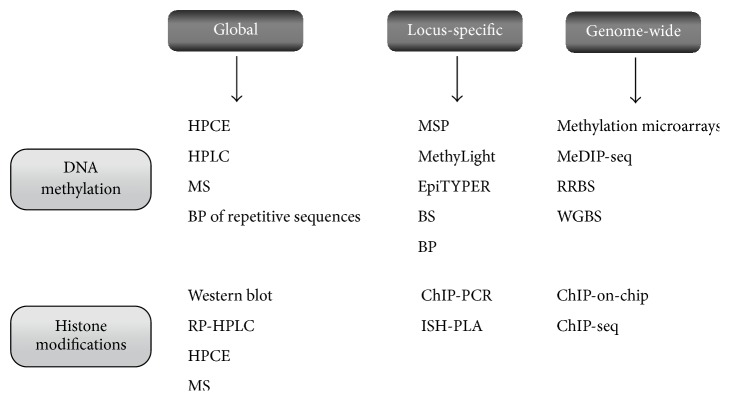
Summary of methods for DNA methylation and histone modification analysis. Different approaches, depending on whether global, locus-specific, or genome-wide analyses, are performed. HPCE: high-performance capillary electrophoresis; HPLC: high-performance liquid chromatography; MS: mass spectrometry; BP: bisulfite pyrosequencing; MSP: methylation-specific PCR; BS: bisulfite sequencing; MeDIP-seq: methylated DNA immunoprecipitation sequencing; RRBS: reduced representation bisulfite sequencing; WGBS: whole genome bisulfite sequencing; RP-HPLC: reversed-phase high-performance liquid chromatography (RP-HPLC); ChIP: chromatin immunoprecipitation; ISH-PLA:* in situ* hybridization and proximity ligation assay.

*DNA methylation* is the best-known epigenetic mechanism in mammals, not only because it was the first discovered, but because it is also easier to measure [12, 13] (Figure 1). It is in fact one of the principal epigenetic events in the human genome and an important regulator of transcriptional activity, genomic imprinting, development, and tumorigenesis [14–16]. Methylation consists in the addition of a methyl group at the carbon 5 position of the cytosine ring to obtain 5-methylcytosine. It is a postreplication modification that appears nonuniformly in the human genome, which contains unmethylated areas intercalated by methylated regions [17, 18]. Methylation occurs predominantly in cytosines of CpG dinucleotides. In vertebrates, around 70–80% of all CpGs are methylated, specifically in repressive heterochromatin regions and in repetitive sequences, such as retrotransposable elements [19, 20]. These CpGs are asymmetrically distributed into CpG-poor regions and CpG-dense regions called “CpG islands” which are located in the promoter regions in approximately 60% of genes and are usually nonmethylated [21, 22].

In general, CpG island methylation is related to gene silencing. DNA methylation promotes the binding of methyl binding proteins (MBPs), which mediate the recruitment of transcriptional repressors [23]. When this epigenetic change occurs in CpG islands located in promoter regions, the silencing can affect important cellular pathways involved in the development of multiple diseases such as cancer [24]. However, it is known that methylation is more dynamic in CpG shores, the genomic regions that are delimited as the 2 Kb regions flanking CpG islands, and in CpG shelf regions, that is, those beyond the CpG shores, 2–4 Kb beyond the CpG islands. Silencing by DNA methylation can also occur in these adjacent regions, where differences in methylation patterns have been found which are related to tissue specific differentiation and cancer [25–28]. Methylation can be catalyzed by two important groups of DNA methyltransferases (DNMTs) [29]: in the first group is DNMT1, which is essential in cell proliferation and ensures the maintenance of DNA-methylation patterns during DNA replication through the methylation of hemimethylated CpGs [30]. The second group includes DNMT3a and DNMT3b, which are required for* de novo* methylation and for establishing methylation patterns in early embryos and during development [31]. Their activity is also known to be necessary for the maintenance of methylation patterns in somatic cells [32].

DNA methylation is crucial for physiological development, playing a fundamental role in gene expression programs during cell fate differentiation [33, 34]. Several studies have identified global DNA hypomethylation related to aging as well as tumoral processes [35–37]. This global decline of methylation levels has also been described as being associated with gene-specific hypermethylation [38, 39]. An illustration of these similar methylation patterns observed in both cancer and aging processes can be seen in relation to the promoter region of the estrogen receptor gene (ER), which is specifically hypermethylated in colon cancer and old individuals compared to normal patients and young people, respectively [39]. DNA methylation is also related to genomic imprinting, X chromosome inactivation mechanisms in females, and the silencing of foreign nucleic acids [40].

*Histone modifications* are another epigenetic mechanism that has been well studied and linked to development processes and aging [41, 42]. Chemical changes in histone protein residues are known as posttranslational modifications (PTMs). These variations usually affect protein functions or gene expression, ultimately impacting on biological processes [43]. PTMs involve reversible modifications commonly located in the N-terminal tails of histones, such as acetylation, methylation, phosphorylation, and ubiquitylation [44, 45]. These marks taken in combination form a “histone code,” which establishes a regulatory mechanism of chromatin dynamics which affects affinity in protein interactions, protein-DNA binding, and gene transcription [46, 47]. They also play an important role in DNA repair [48], DNA replication, and chromatin compaction [43]. These PTMs are “written” by different families of enzymes; for example, histone-acetyltransferases (HATs) and deacetylases (HDACs) are involved in histone acetylation, while histone methyltransferases (HMTs) catalyze the transference of up to three methyl groups onto the lysine and arginine amino acids on histones, predominantly H3 and H4 [49]. The most common PTMs are in fact the acetylation and methylation of lysines on histone tails [46, 50]. Acetylation of lysine by its corresponding enzymes is linked to transcription activity [51]. However, histone methylation can be related to either gene activation or repression depending on which residues are affected; for example, H3K4me3 and H3K36me3 are active markers while H3K27me3 and H3K9me3 are both related to gene repression and heterochromatin [43, 46, 50, 52, 53].

Apart from the groups of enzymes above, there also exist proteins with specific domains that are able to identify these combinatorial PTMs. For example, proteins with bromodomains or chromodomains recognize and bind to acetylated lysines and methylated lysines, respectively [54, 55]. These effector proteins read and interpret histone marks [46, 56] and are involved in the regulation of transcriptional response. Greater knowledge of these protein functions has increased interest in their study as a promising new class of drug targets for a wide range of human diseases and for therapeutic development. For example, the development of BET (bromodomain and extraterminal) inhibitors selectively modulates the expression of genes involved in cell growth and invasion and antiapoptotic activity [57–59] associated with tumoral progression.

In some cases these epigenetic modifications are the result of environmental factors and may have important roles in the development of normal and pathological processes [60, 61]. In cancer disease, studies have identified PTMs associated with carcinogenesis. For example, loss of acetylation at Lys16 and trimethylation at Lys20 of histone H4 were found to be related to neoplastic processes [62]. Alterations in histone-modifying enzymes have been identified as the main cause of these changes in many cases [63–66] and as a result they have started to gain importance as a therapeutic target in recent years [67–69]. Histone PTMs are implicated not only in cancer, but also in a wide range of pathologies related to chronic diseases, such as diabetes and obesity [70], renal disease [71], and neuropathologies [72].

Although* ncRNAs* are not as well studied as DNA methylation or histone PTMs, they are attracting attention because of their important role for normal cell development and function, as well as in relation to disease. ncRNAs can be divided into short, intermediate, and heterogeneous group of long RNAs [73, 74]. ncRNAs such as micro (miRNAs) or long antisense noncoding RNAs (lncRNAs) have recently been acknowledged to play a role in epigenetics, and furthermore, they may be affected by certain environmental factors [75, 76]. An miRNA is a single-stranded RNA molecule, 21-22 nucleotides long, which has a completely different function compared to the most frequent single stranded RNA molecules such as messenger RNA (mRNA). In addition, miRNAs are not translated into protein and they are in part complementary to various mRNA molecules, to which they are able to bind to facilitate their elimination and subsequent gene expression [73]. lncRNAs are considered another epigenetic regulation mechanism of protein-coding gene expression, and they recruit histones and chromatin related proteins to specific sites [77, 78]. Loss of their activity may interfere with the transcription of various genes [79] and the aberrant functioning of lncRNA causes the deregulation of genes that are involved in a number of diseases [80]. When silencing affects tumor suppressor genes, it can contribute to cancer progress. One example is ANRIL, an lncRNA involved in the silencing of different tumor suppressor genes, such as NK4n/ARF/INK4a, p16/CDKN2A, and p15/CDKN2B, which are related to cell cycle and senescence [81, 82].

The epigenetic mechanisms described above are connected and integrated to regulate gene expression and cell fate through a complex scenario whereby the functions that they regulate interact [83]. For instance, the expression of miRNAs is usually controlled by DNA methylation, and epigenetic alterations can lead to disease phenotypes [84, 85].

## 3. Causes of Epigenetic Changes during Development, Adult Life and Aging

Epigenetic marks constantly change throughout life. Some of these changes are programed and play important roles in the different stages of development, but the mechanisms involved are still not fully understood [86–88]. Intrinsic, or genetic, factors are of great importance in regulating certain epigenetic changes which occur over time. In support of this, the epigenomes of monozygotic twins are known to be more similar than those of dizygotic twins [89]. One important example of an intrinsic epigenetic mechanism which is programed during life is female puberty, which is initiated by the secretion of gonadotrophin-releasing hormone (GnRH) and the activation of the hypothalamic-pituitary-gonadal (HPG) axis [90]. Currently a few studies have related epigenetic mechanisms to female puberty regulation, supporting the notion that the activation of neuroendocrine pubertal components is mediated, at least in part, by epigenetic mechanisms [90].

Besides the intrinsic epigenetic changes, there are some epigenetic alterations that take place apparently by chance, without evident biological function [7, 91]. The distinction between stochastic changes and environment-mediated changes is sometimes very difficult to establish as stochastic changes can potentially be modulated by both intrinsic and extrinsic (environmental) factors [7, 87]. Although this influence of environmental factors has been widely reported [92], the manner of the interplay between the environment and the epigenome remains largely unknown. Apart from intrinsic and stochastic factors that may activate epigenetic mechanisms, the main factors modulating these mechanisms are extrinsic factors like environmental situation. Many studies have provided support for the theory that environmental pressure during the developmental stages of early life (both prenatal and in childhood), such as nutritional status or exposure to toxic compounds, can affect epigenetic developmental programing. This has given rise to the term “developmental origin of health and disease” (DOHaD), which proposes a broad range of environmental phenomena in the early stages of life that may increase disease susceptibility during adult life [93]. Consequently, when studying the influence of the environment, it is necessary to take into account two dissimilar scenarios where the environment has an impact: embryonic development and during lifetime. The first scenario is the most susceptible to any external influence due to the high number of cell division events and the critical epigenetic changes that take place during cell differentiation [94]. Furthermore, the effect of any epigenetic change in an undifferentiated cell can be transmitted and amplified to future cell populations.

During embryonic development, environmental conditions can be modulated by two different principal factors: the lifestyle of the mother, which necessarily implies embryo exposures, and the anatomical/phenotypic circumstances of the mother, such as size of the uterus and placenta. It is a period where nutrient supply and chemical exposure have a critical influence on the epigenome [88] and any resulting epigenetic dysregulation could lead to disease development during adult life. As well as mother influence, studies in rats and mice have associated paternal obesity with a reduction in the implantation rate of the blastocyst [95–97].

In relation to the adult epigenome and how it is affected by the environment, despite an ample literature in the field, the molecular mechanisms implicated are still only poorly understood [94]. What is well known, though, is that the influence of external factors on the genome depends on the tissue type involved. For instance, it is easy to see that UV radiation could have a harmful effect on the skin, but rather less obvious to see that muscle can also be affected. In addition, when an alteration affects adult stem cells the consequences are likely to be more serious than when differentiated cells are involved [88]. And most importantly, if the germline is affected, reproductive disorders might result and even the possibility of transgenerational inheritance of the epigenetic alterations [5].

For the purposes of this review we will only consider those external factors which can affect the epigenome. We will discuss how chemical and physical environmental stressors, diet, life habits, and pharmacological treatments can alter the epigenome during lifetime and how these alterations can determine healthy and disease phenotypes.

### 3.1. Chemical and Physical Environmental Stressors

Epigenetic marks can be affected by exposure to metals, air pollution, benzene, organic pollutants, and electromagnetic radiation [98]. Chemical and xenobiotic compounds in water or the atmosphere are other potential environmental stressors capable of changing epigenetic status. During embryonic development, the effect of exposure to environmental pollutants seems to have an even more crucial effect on the epigenome and increases the risk of developing disease in the F_1_, F_2_, and F_3_ generations [99]. In this section we will describe several studies showing how several stressors such as metals and air pollutants can affect the epigenome, which in turn is related to the appearance of certain diseases.

It is known that environmental exposure to a variety of metals, such as arsenic, mercury, nickel, lead, and cadmium, has several impacts on human healthy. Many recent studies suggest that alterations in epigenetic mechanisms could play a key role in the molecular mechanisms involved in the metal exposure-related diseases. Arsenic (As) is considered the most widespread metal in the environment. It is present in rocks, soil, water, insecticides, and airborne particles, among other things [100, 101]. Chronic arsenic exposure is related to many health problems such as skin lesions, neuropathy, depression, cardiovascular diseases, and various kinds of cancers [102–106]. Experimental analyses have found DNA methylation changes after arsenic exposure, both global [107, 108] and gene-specific [109]. In addition, arsenic exposure is capable of inducing H3K4me3 and H3k9ac enrichment [110–113] and H3K27me3 decrease due to alterations in histone-modifying enzymes [113]. During human development, arsenic exposure has also been associated with changes in DNA methylation patterns of cord bloods during the prenatal period [114, 115] and with gene-specific DNA methylation changes in white blood cells [116] and in the placenta [117]. In adults, arsenic concentrations have been associated with LINE-1 DNA hypomethylation in different population-based studies [118, 119]. Furthermore, it has been found that it is able to induce DNA methylation changes in a gene-specific way. For instance, high arsenic exposure was found to be related to DNA hypermethylation of the tumor suppressor genes* p16* [120] and* RASSF1A* [121]. In Bangladeshi adults, a link was found between arsenic exposure and global PTM changes, positively correlated with H3K9me2 and inversely with H3K9ac [122]. The relationship between arsenic exposure and epigenetic alterations remains unclear, but an* in vitro* study has pointed to chronic arsenic exposure inducing loss of DNA methylation through SAM depletion [123].

Cadmium (Cd) is a chemical element which is widespread in the environment, in byproducts of industrial processes, contaminated water, or soil, and it also has many common industrial uses, as a component in battery production, for example. Furthermore, cadmium exposure is mainly the result of diet, principally through cereals and vegetables, and also smoking. It causes many health problems, such as cancer, increased risk of bone fracture, kidney damage, and probably impaired early-life development [124, 125]. Epigenetic alterations could be implicated in cadmium toxicity mechanisms during embryonic development and lifetime. For instance, a set of genes related to transcriptional regulation control and apoptosis showed DNA methylation changes associated with maternal cadmium concentrations [126]. In adults, urinary cadmium concentrations of women were inversely associated with LINE-1 methylation and also negatively associated with* DNMT3B* expression [127].

Lead (Pb) is a poisonous heavy metal used in building construction, batteries, and consumer products, among other uses [128]. Exposure to lead is a great risk for human health, affecting a variety of fundamental molecular processes [129]. For instance, maternal exposure was shown to result in neurodevelopmental deficit [130] and reduced intelligence of the child [131], and* in vitro* Pb exposure of hESCs induced changes in the methylation status of genes involved in neurogenetic signaling pathways [132]. In humans, LINE-1 and Alu repeat DNA hypomethylation were correlated with Pb levels in umbilical cord blood [133, 134], and it was shown that early-life Pb exposure caused gender-specific changes in DNA methylation in dried blood spots [135]. In adults, male Pb levels were related to LINE-1 DNA hypomethylation [134, 136]. Another study, in women, has shown* COL1A2* promoter DNA hypomethylation with high exposure to lead [137].

Mercury (Hg) is a reactive metal whose physiological activity is unknown. Products containing mercury include batteries, fluorescent bulbs, medical products, dental amalgams, thermometers, and thermostats. However, humans are mainly exposed to mercury through fish and shellfish, which tend to concentrate mercury in their bodies. Some mercury-related health outcomes are immunotoxic effects, cardiovascular disease, cancer, and kidney disease [138–140].* In vivo* studies in rats have revealed that prenatal exposure to mercury produces a reduction in neural cell proliferation, which is associated with DNA hypomethylation [141]. In humans, a recent study showed that* in utero* exposure to mercury, even at low levels, produced changes in DNA methylation [114]. Furthermore, studies have hypothesized that prenatal mercury exposure can change the proportion of immune cells in cord blood through DNA methylation changes [114, 142]. In adult women, there is an increase in the DNA methylation of the promoter region of the tumor suppressor gene GSTM1 following high levels of mercury exposure [137]. Another study in male dental professionals found a correlation between SEPP1 DNA hypomethylation and hair mercury levels [143].

Nickel (Ni) is a metal that is widely occurring nowadays. It is used in jewelry, coins, batteries, and medical devices, among other things. The International Agency for Research on Cancer (IARC) has determined that some nickel compounds are carcinogenic to humans (mainly linked to respiratory cancers), but the state of knowledge of the molecular mechanisms implicated is low and further research is required.* In vitro* experiments have shown that this compound is able to increase global levels of H3k4me3, H3K9me1, and H3K9me2 through demethylase inhibition [110, 144, 145]. In adults, nail concentrations of nickel were positively correlated with LINE-1 DNA methylation levels [118].

Besides alterations of DNA methylation or/and histone modifications, numerous changes in miRNA profiles have been associated with exposure to different metals, among them mercury, arsenic, and cadmium [146], and a negative correlation between mercury and lead levels and various miRNAs has been found in cervical swabs from pregnant women [147].

In addition to metals, air pollutants can also affect the epigenome. In adults, exposure to atmospheric pollutants, especially those which are traffic-related, has been associated with a reduction in lung function and with lung cancer, which could be due to changes in the DNA methylation of inflammation and immunity genes, as well as of repetitive elements [148, 149]. The effects of exposure to particulate matter (PM) on global and gene-specific methylation in workers in the steel industry, which has high levels of PM exposure, have also been investigated. Inducible nitric oxide synthase (iNOS) was found to have significantly decreased promoter methylation after PM exposure [150]. Moreover, exposure to black carbon, a marker of traffic particles, has also been found to be associated with aberrant global DNA methylation and to be related to cardiovascular disease [151]. In some cases, differential miRNA expression produced by environmental exposure, including air pollution, may be associated with human diseases. In adults, alterations of miR-9, miR-10b, miR-21, miR-128, miR-143, miR-155, miR-222, miR-223, and miR-338 associated with air pollution have been observed in various studies [152]. Both upregulation and downregulation of several miRNAs caused by diesel exhaust particle (DEP) exposure have been associated with human airway diseases [153].

Asbestos can also alter the epigenome. Exposure in adults induces malignant pleural mesothelioma (MPM), although the pathogenic mechanisms implicated in the tumor transformation are not well characterized [154]. Many studies have related asbestos exposure to promoter DNA methylation of many tumor suppressor genes such as* APC*,* CCND2*,* CDKN2A*,* CDKN2B*,* ESR1*,* HPPBP1*,* RASSF1*,* SLC6A20*,* SYK*, and* ZIC1* in MPM [155–157]. Along with asbestos, benzene is a major immunosuppressive agent. Bollati and collaborators found that low benzene exposure is able to induce peripheral blood DNA methylation changes, such as a decrease in LINE-1, AluI, and MAGE-1 methylation and* p15* hypermethylation. These changes could increase the risk of developing acute myelogenous leukemia [158, 159].

Endocrine disruptors are chemical pollutants which at certain doses can affect the endocrine system and produce adverse developmental, reproductive, neurological, and immune effects. Many compounds act in this way, such as pesticides (DDT and methoxychlor), fungicides (vinclozolin), herbicides (atrazine), industrial chemicals (PCBs, dioxins), and plant hormones (phytoestrogens) [5, 160], although those most frequently affecting mammalian organisms are plastics, specifically bisphenol A (BPA) and the phthalates. The impacts of exposure to both these plastics on human health have been widely reviewed and reported by the National Toxicology Program-Center for the Evaluation of Risks to Human Reproduction [161, 162]. There is an ever increasing potential health risk associated with exposure to these two chemicals due to their extensive use in the manufacturing of polycarbonate plastics [163]. Effects of exposure include a decrease in female fertility and an increase in cancer susceptibility [164, 165]. BPA is a carbon-based synthetic compound employed to make certain consumer goods, such as CDs, DVDs, plastic bottles and containers, and the epoxy resins that line metal food and drink cans. Global hypomethylation has been found to result from BPA exposure in animal models such as* Agouti* mice [166, 167], in human spermatozoa [168], and on chromosome X in prepubescent girls [169].* In vitro* study has related BPA treatment to an increase in promoter DNA methylation levels of* LAMP-3* (lysosomal-associated membrane protein 3), Nsbp1 (nucleosome binding protein-1), and Hpcal1 (hippocalcin-like 1) genes [170, 171]. An* in vivo* study in rats has shown an increase in PGC-1*α* DNA methylation related to induction of cardiomyopathy [172]. BPA has also been found capable of altering the expression and DNA methylation of many imprinted genes, including* Snrpn*,* Ube3a*,* Igf2*,* Kcnq1ot1*,* Cdkn1c*, and* Ascl2* [173, 174]. Apart from the DNA methylation changes produced by BPA, it also results in an increase in EZH2 activity and histone H3 trimethylation levels [170, 175].

The effect of electromagnetic radiation on the epigenome has also been investigated. Prolonged exposure to ultraviolet (UV) light is associated with the development of various skin lesions and cutaneous malignances [176, 177]. It is well known that solar UV radiation is involved in oxidative stress (3) [178], immune system alterations [179], and gene mutation and DNA damage (6) [180]. Some studies have also found changes at the epigenetic level associated with chronic exposure to ultraviolet radiation. One study found aberrant DNA hypermethylation linked to increased DNA methyltransferases activity and hypoacetylation of H3 and H4 in UV exposed epidermal cells in mouse models [181]. These epigenetic modifications could be related to the transcriptional silencing of certain tumor suppressor genes and thus result in stimulating skin tumor formation. The identification of aberrant methylation in genes such as Cip1/p21 and p16 INK4a [182], genes of the cadherin and laminin families [183], and the inactivation of the RB1/p16 and p53 pathways in cutaneous squamous cell carcinoma [184], as well as other studies, supports the notion that important epigenetic changes are mediated by chronic sun or UV radiation exposure. These and other findings have allowed the identification of genes with aberrant methylation that could be used as molecular markers which are commonly perturbed in malignant skin lesions (10) [185–187], and the development of new therapies to reverse those epigenetic changes associated with UV radiation. In addition to the clinical chemopreventive agents which exist for blocking the aberrant methylation or histone deacetylation caused in many tumorigenesis processes, there are also other natural compounds found in a variety of different foods which have a photoprotector effect. Several experimental studies in* in vivo* models have shown the beneficiary effect of proanthocyanidins from grape seeds [188] and polyphenols from green tea [189, 190], among others, in countering photocarcinogenesis.

### 3.2. Diet

**Figure 2:**
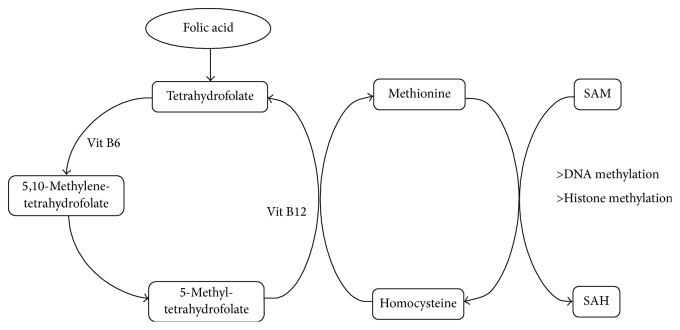
Scheme of methionine pathway. A metabolic pathway which represents the synthesis of SAM through folate intake. SAM: S-adenosylmethionine; SAH: S-adenosylhomocysteine.

Probably the most widely described example of diet affecting epigenetic marks is the study of the intake of folate and other methyl donors during prenatal stages. Vitamin B9, or folic acid, used in the synthesis of tetrahydrofolate, cannot be synthesized* de novo* by the human body; hence it needs to be supplied from the diet. Furthermore, vitamin B6 functions as a cofactor in the synthesis of 5-methyltetrahydrofolate, the methyl donor for the B12-dependent remethylation of homocysteine to S-adenosylmethionine (SAM), and the methyl donor group for DNA and histones which is necessary to maintain methylation levels (Figure 2). The methyl donor suppliers can affect the epigenome in a global manner and also in a locus-specific way [87, 191], and lack of folic acid nutritional supplement contributes to the induction of cancer in animal models of disease [192]. The best example is the influence of maternal diet on the murine* Agouti* gene (A^vy^). This gene is responsible for determining if mouse coat color is banded (*Agouti*) or solid (non-*Agouti*) and is regulated by the DNA methylation status of the intracisternal A-particle (IAP) retrotransposon located in the promoter region of the* Agouti* gene [193, 194]. When it is methylated, a mouse's coat has a normal appearance (solid color), but when the retrotransposon is unmethylated, the coat is banded or yellow and the mouse will have an increased risk of cancer and diabetes. The availability of methyl donors before and during pregnancy was found to increase the methylation status of the promoter* Agouti* gene in future offspring [195].

In humans, there are some studies showing the effects of diet and food availability on the epigenome and how these epigenetic changes could be involved in the appearance of several diseases in adulthood [191, 196]. A clear example of these relationships is reflected in the results of studies of the offspring of pregnant women during the Dutch famine of 1944-1945, during the last period of the Second World War [191, 197]. Another representative example showing the effects of nutrition comes from a study in Gambia showing differences in the epigenomes of children conceived during the nutrient-poor rainy season compared to those conceived in periods where the nutritional intake of the mother is better [196].

Although dietary folate deficiencies are most obvious when they take place during embryonic development, during adult life the amount of folate intake in the diet has been found to be related to epigenetic status in mammals [198, 199], including humans [200], and has also been related to methylation changes in colon cancer [201] and hyperhomocysteinemia [202]. Other dietary methyl donors are methionine, which is involved in the metabolic pathway of SAM and has been related to epigenetic-dependent hepatic disorders [203], and selenium, which is a dietary supplement capable of modifying the epigenetic status of prostate cancer cells and reducing both DNA and histone methylation levels and which has been suggested to improve cancer prevention through the activation of silenced genes [204]. In addition it has been described that deficiency in vitamin B12, another methyl donor, plays an important role in the adipocyte metabolism, and its deficiency leads to increased total cholesterol by limiting S-adenosylmethionine [205].

In relation to adults, caloric restriction (CR) is a dietary regimen based on a reduction in caloric intake which many studies have related to lifespan extension in various eukaryote organisms [206–212]. There are numerous studies supporting the notion that CR protects against many different diseases related to aging due to a reduction in oxidative stress and regulation of metabolic pathways [213–216]. The molecular mechanisms involved in this regulation are varied, and epigenetic marks could play an important role in the processes [217, 218]. For example, it is thought that CR might attenuate the epigenetic changes occurring during the progress of aging [219–221].

Bioactive dietary compounds such as polyphenols can alter developmental plasticity through the generation of epigenetic changes and may also play a role in health and disease. In the last two decades much research has been focused on the mechanisms that could be responsible, at least in part, for the relationship between regular consumption of such bioactive compounds and the changes that may be produced in the epigenome and result in improvements in health and aging. This has given rise to the novel field of study known as epigenetic influence of nutrition [222]. Since numerous bioactive dietary compounds appear to have the potential to promote health or prevent diseases, such beneficial dietary supplements could be used to complement other therapies. Polyphenols, a structural class of organic chemicals characterized by the presence of large multiples of phenol structural units, are necessary in the human diet and can be found in fruits and vegetables [223]. Plant-origin polyphenols can be classified into different groups based on their chemical configuration and include flavonoids, stilbenes, phenolic acids, benzoquinones, acetophenones, lignins, and xanthones [224]. Some authors estimate that more than 8000 distinct dietary polyphenols exist, including resveratrol (found in grapes), epigallocatechin-3-gallate (EGCG, in green tea), sulforaphane (SFN [1-isothiocyanato-4-(methylsulfinyl) butane], in broccoli), and curcumin (in turmeric) [225]. Although more studies are needed, the potential of green tea and broccoli in cancer chemoprevention seems to be mediated by epigenetic mechanisms, including DNMT and HDAC activity inhibition [226, 227].

### 3.3. Healthy and Unhealthy Habits

Although unhealthy lifestyle habits could be considered as a kind of environmental stressor, the fact that they depend on personal decisions has led us to describe them in a separate section.

It is well known that maternal tobacco smoke exposure (MTSE) is one of the most important risk factors during pregnancy for many diseases such as asthma, cancer, obesity, and type II diabetes [228–231]. MTSE is known to produce epigenetic changes that can affect birth-weight and fetal programing [232], specifically DNA methylation: there is global DNA hypomethylation and an increase in DNA promoter specific methylation in children exposed to prenatal smoking compared to children who were not [233]. In adults tobacco use has been related to an increase in promoter gene-specific DNA methylation, which in turn is linked to increased predisposition to diseases such as cancer [234–237]. To investigate in more depth the effect of tobacco smoking on DNA methylation, researchers have performed genome-wide DNA methylation analyses with the Illumina 450 K BeadChip. As a result, tobacco use has been related to changes in DNA methylation of CpG sites related to the development and function of the cellular, cardiovascular, detoxification, hematological, immune, tumorigenic, and reproduction systems [238–241]. Apart from DNA methylation, smoking also affects proper histone regulation across the polycomb repressive complex, coinciding with decreased H4k16ac and increased H3k27me3 [242]. Many substances contained in tobacco can also affect miRNAs. During development, a decrease in the expression of miRNAs, such as miR-16, miR-21, and miR-146a, have been related to nicotine and benzoapyrene exposure in smoking mothers [243]. In adult smokers miR-218 has been shown to be downregulated in bronchial epithelial cells [244]. Also, smoking has been associated with alterations in the expression of miRNAs such as miR-21, miR-34b, miR-125b, miR-146a, miR-223, and miR-340 [152]. Moreover, cigarette smoke condensate (CSC) causes aberrant overexpression of miR-31 in lung epithelium, and it could act as an oncomir promoting pulmonary carcinogenesis [245].

High alcohol consumption is also widely recognized to have many negative effects which lead to a deterioration in an individual's health. Alcohol can interfere with methionine metabolism through the inhibition of methionine synthase and, as a consequence, the long-term use of alcohol could lead to a decrease in the hepatocyte level of SAM [246]. In adult life, hypomethylation in LINE1 has been related to alcohol consumption in some tumors [247, 248]. Other studies have revealed that the use of alcohol alters DNA methylation patterns in hepatocarcinogenesis and neural stem cell differentiation [249, 250]. In addition, miR-125 and miR-126 downregulation has been observed in alcohol consumption related to hepatocellular carcinoma [251, 252].

Apart from DNA methylation and miRNA changes, ethanol induces gene activation through an increase in histone H3 and H4 acetylation and H3k4me3 [253–256], which may lead to immune system dysfunction [257]. Interestingly, epigenetic changes due to ethanol seem to be different depending on whether there is chronic or binge ethanol intake [253]. Furthermore, prenatal alcohol exposure also significantly affects the correct development of the fetus, including altering PcG/TrxG programing [258].

Substantial stress during early life can be a risk factor in the initial appearance of symptoms for individuals susceptible to bipolar disorder and other mental disorders [259]. Many studies have reported a relationship between early life stress and the aberrant DNA methylation of many genes such as the glucocorticoid receptor gene [260, 261] and the serotonin 1A receptor [262]. Also, stress is able to produce changes in histone modifications, such as increased levels of H3K4me3 and reduction of H3K9me3 levels in the dentate gyrus [263]. In this regard, there is an interesting study showing how prenatal maternal stress, generated by a natural disaster, was related to changes in DNA methylation patterns of blood cells, which could have an effect on the immune function of the offspring [264].

Physical exercise enhances or maintains physical fitness and is beneficial for human health in a number of ways. Little is known about the molecular mechanisms responsible, but several studies have shown that epigenetics is related to the effects of exercise on human health, since epigenetic changes in germ cells, skeletal muscle, and brain have been observed following a period of exercise [265–267].

### 3.4. Pharmacological Factors

Pharmacological treatment can also induce genome-wide epigenetic changes. Sodium valproate (VPA), a small fatty acid [268], has been widely studied. It is used to treat epilepsy, bipolar disorder, serious depression, migraine, and schizophrenia, as well as being used in cancer treatment and as a complementary treatment for latent HIV infection [269, 270]. Because of VPA's global HDAC inhibitor effect [271], it could possibly generate the expression of some undesirable genes, and its side effects remain to be fully demonstrated.

Diethylstilbestrol (DES) is a “synthetic estrogen” which has been used for many years during pregnancy to prevent miscarriages and other pregnancy disorders but has been found to be associated with an increased risk of breast cancer and vaginal and cervical adenocarcinoma [272, 273]. It has been suggested that these side effects are mediated by epigenetic mechanisms, since it has been found that DES neonatal exposure in mice was related to decreased DNMT expression and alterations in DNA methylation in the mouse uterus [274]. In addition, it has been described that DES exposure in breast epithelial cells produces upregulation and downregulation of various miRNAs [275]. Moreover, miR-21 was consistently downregulated by DES exposure in MCF-7 breast cancer cell line [276].

Apart from these two drugs, there are many different medicines used on a daily basis that have recently been found to have epigenetic activity. For example, procaine, a local anesthetic, is now known to induce DNA demethylation [277]. And several antibiotics have also been implicated [278], such as pyrazinamide, a classic antituberculosis drug found to alter DNA methylation in the liver of treated rats, along with* LINE-1* hypomethylation and GSTP and* p16(INK4A)* promoter hypermethylation, all of which may be a side effect of its hepatic toxicity [279]. In addition, doxorubicin, an anthracycline antitumor antibiotic, has been found to inhibit DNMT1 and can induce apoptotic cell death [280]. miRNA profiles may also be altered by many different drugs used in therapy, for example, all-*trans*-retinoic acid in acute promyelocytic leukemia [281] and gemcitabine [282] and cisplatin [283] in ovarian cancer. Most importantly, perhaps, attention should be paid to the effects of medication on the germ cell epigenome and the potential transmission of epigenetic alterations to offspring. An example is where aberrant DNA methylation patterns were found in the sperm of patients treated with temozolomide, a chemotherapy drug used as a treatment for high-grade glioma [284].

## 4. Conclusions

The mammalian epigenome changes throughout embryonic development and with aging. Some of these changes are genetically programed and others take place without any apparent function, though the molecular mechanisms involved are still to be elucidated. Moreover, what part of these changes is due to the interplay of environment with the epigenome and which is the result of individual genetics remain unknown. Further research needs to be focused on attempting to understand the causes of these changes in order to prevent the onset of diseases. In addition to all the environmental factors affecting the epigenome described in this review, we should take into account increased exposure to the nanomaterials and nanoparticles present in many everyday consumer goods, and consequently the study of the possible effects of this on the epigenome and human health will be a future, or rather is a current, challenge. The emergence of next-generation technologies will help us to answer some of these questions in the coming years.
